# Effects of Phonological Training on the Reading and Reading-Related Abilities of Hong Kong Children with Dyslexia

**DOI:** 10.3389/fpsyg.2017.01904

**Published:** 2017-10-31

**Authors:** Li-Chih Wang

**Affiliations:** Department of Special Education and Counselling, Education University of Hong Kong, Tai Po, Hong Kong

**Keywords:** Chinese, dyslexia, onset-rime-level phonological awareness, phonological awareness training

## Abstract

This study aimed to investigate phonological awareness training by examining outcomes among Chinese children who learn Chinese without phonetic system training. Fifty-six Hong Kong children from the 3rd to 6th grades were recruited. Two-thirds of the children had been officially identified as dyslexic by the local government, and the remainder were considered high risk for dyslexia. The children were divided equally into a control group and an experimental group, with the groups matched as closely as possible by age and gender. Children in the experimental group were trained by onset-rime-level phonological training. The training lasted ~3 weeks, with 15 daily sessions lasting ~20 min each. Our results indicated that children in the experimental group made significant improvements in Chinese character reading, onset awareness, rime awareness, and rapid naming after training. The association between phonological awareness and Chinese character reading, especially the association between rime awareness and Chinese character reading, also changed after training. The benefits of phonological awareness training were more obvious for children younger than 10 years old. The results of the present study can be extended to provide another approach to Chinese learning for children suffering from reading difficulties who are not responding to the usual approach in their region.

## Chinese children with dyslexia

Dyslexia, one of the most common neurobehavioral problems affecting children (Shaywitz et al., 2004), is a learning disability that is characterized primarily by difficulties in spelling and decoding (Lyon et al., 2003). Previous investigations have found that reading difficulties or dyslexia affect ~5–17% of children in the United States (Shaywitz et al., 1990). Similar numbers have been reported in other countries with alphabetic (e.g., Korhonen et al., 2014) or non-alphabetic languages (e.g., Chan et al., 2007). These specific learning disabilities are marked by difficulties in the development of cognitive abilities and reading-related skills (Catts, 1991). A great deal of evidence has demonstrated that due to this reading-related deficit, dyslexic children perform significantly worse than those without dyslexia, mainly in phonological processing (Ziegler and Goswami, 2005), including phonological awareness (the awareness of the phonological structure, or sound structure, of words) (e.g., Bruck, 1992), and rapid naming (the index of how quickly individuals can name stimuli aloud) (e.g., Lervåg and Hulme, 2009). Based on recent evidence, slower rapid naming at early ages is considered an early indicator of reading problems in the future (for a review, see Norton and Wolf, 2012).

A growing body of evidence indicates that the behavioral markers of children with dyslexia in non-alphabetic languages such as Chinese differ from those of children with dyslexia in alphabetic languages. Furthermore, the cognitive profile of Chinese dyslexia is more complicated. The basic graphic unit in Chinese is a character. Approximately 80% of Chinese characters are compound characters (Chung and Leung, 2008) that are constructed by a number of stroke patterns, also known as radicals (Chung et al., 2008). Therefore, it is not surprising that reading Chinese characters relies heavily on readers' visual processing skills (Liu et al., 2015). Individuals who have difficulty reading Chinese have been demonstrated to have deficits in visual-orthographic knowledge (Ho et al., 2002, 2004).

Although Chinese children with dyslexia have been consistently found to have relatively poor orthographic knowledge, rapid naming, morphological awareness, and phonological memory (e.g., Ho and Lai, 1999; Ho et al., 2002; Chan et al., 2006; Shu et al., 2006), evidence regarding whether their phonological awareness is worse than those without dyslexia remains controversial (e.g., Huang and Zhang, 1997; Lee and Ko, 2007). This inconsistency can be attributed not only to the written script of Chinese characters but also to the instructional method used (Chen and Yuen, 1991).

### Diverse instructional methods in chinese and their influences on phonological awareness

In general, children learn to read in two main ways in the Chinese context. On the one hand, children from Hong Kong or Macau learn to read Chinese characters using the look-and-say method without a system of phonetic symbols to label their pronunciations. In other words, Hong Kong or Macau teachers are accustomed to instructing children through the rapid retrieval of the names of Chinese characters, which may strengthen Hong Kong or Macau children's rapid naming abilities when reading (Ho, 2010). On the other hand, children in mainland China and Taiwan learn to read Chinese characters from a system of phonetic symbols (mainland China: pinyin; Taiwan: Zhuyin Fuhao) that includes not only visual cues for sounds but also systematic training that is highly similar to phonological awareness training (Lin et al., 2010).

Zhuyin Fuhao is a typical approach used in Taiwan textbooks to assist early primary school-aged students in recognizing the pronunciation of characters. There are 21 onsets, three medials, 13 rimes, and four tones in this system, and no more than three symbols are allowed. The symbols in Zhuyin Fuhao are fabricated upon this system, so Taiwanese students are required to memorize all symbols at the beginning of primary school. In mainland China, pinyin plays a similar function as Zhuyin Fuhao does. However, pinyin is constructed of a systematic alphabet with consistent grapheme-phoneme correspondence that is very similar to the symbols used in English (Stevenson, 1984), with 22 onsets and 38 rimes in the system (Ho and Bryant, 1997). Although the symbols of pinyin and Zhuyin Fuhao are different, the instructional approaches are similar; they begin with simple rimes with a single vowel together with the four tones and then introduce the onsets (Wang and Gao, 2011).

In terms of the instructional approach of both pinyin and Zhuyin Fuhao, the emphasis on grapheme-phoneme conversion may strengthen phonological awareness abilities (Ho, 2010). This view has been supported by abundant evidence.

For instance, some researchers have conducted comparative studies to examine the phonological awareness and reading performance of children who are learning to read with and without phonetic system training. Children who learn to read with phonetic system training tend to have better phonological awareness (e.g., Cheung et al., 2001; McBride-Chang et al., 2004).

Other studies have used longitudinal or cross-sectional designs to demonstrate the effect of early phonetic system learning on subsequent phonological awareness and reading (Shu et al., 2008; Lin et al., 2010). Therefore, it is not surprising that higher percentages of dyslexic children in mainland China have phonological awareness deficits than Hong Kong dyslexic children do (Luan and Ho, adapted from Ho, 2010).

As suggested by the review above, continuous phonological training (i.e., pinyin or Zhuyin Fuhao) may influence students' development of phonological awareness and its importance for reading. This view seems to be consistent with the idea that improved phonological awareness can be transferred to better reading performance despite the diverse nature of Chinese and alphabetic languages (Burgess and Lonigan, 1998). Therefore, it is important to further examine whether the reading of children who have never received phonetic systems training, such as children in Hong Kong or Macau, can benefit from phonological awareness training. This may provide another approach to facilitate reading, especially for those suffering from reading difficulties.

### Effects of phonological awareness training on Hong Kong children

A few studies have demonstrated the effectiveness of phonological awareness trainings (e.g., Ho and Cheung, 1999; Ho and Ma, 2002) for Hong Kong children with dyslexia. The focus of these studies was mainly the orthography-phonology correspondence of Chinese character components. However, the correspondences of Chinese character components and their pronunciations are diverse and rely on a consistent level of pronunciation of sound components (i.e., phonetic radicals) and Chinese characters. In this case, recognition of the location and information of phonetic radicals is crucial. However, the capability to accurately recognize phonetic radicals is highly relevant to the orthographic knowledge of Chinese characters (Ho et al., 2003). Therefore, although the outcomes from the training in the aforementioned studies are effective, it is difficult to claim that positive results are generated by the phonological awareness training.

Recently, Zhou et al. (2012) presented a positive report on this topic. Zhou et al. (2012) provided phonological awareness training, lexical compound training, and homophone training for 88 Hong Kong kindergarteners. Their results indicated that phonological awareness training was less effective than the other two training methods for word reading or vocabulary knowledge. Specifically, phonological awareness training was effective only for Chinese character reading in a pre-test and post-test comparison, although a similar effect was found in the control group. In other words, it is difficult to determine the true effectiveness of phonological awareness training from Zhou et al.'s (2012) study. With respect to their findings, one of the possible reasons for the less effective outcomes is the content of the phonological awareness training. The details are introduced below.

The benefits of phonological training may be reasonably inferred from individuals who learned Chinese with phonological support systems, as in our review above. However, this view has not been confirmed. The nature of different types of phonological awareness may be one probable reason for this lack of clarity. Thus, a clearer understanding of the structure of phonological awareness is important.

### Diversity of phonological awareness

In general, phonological awareness is defined as the ability to detect or manipulate three levels of sounds (Gillon, 2004). In Ziegler and Goswami's (2005) Psycholinguistic Grain Size theory, a hierarchical model of phonological awareness regarding the phonological structure of words, involves phones, phonemes, nucleus-codas, onset-rimes, and syllables. Three of these are emphasized in alphabetic languages (Gillon, 2004; McBride-Chang et al., 2004): the syllable level, the onset-rime level, and the phoneme level. Although these three levels of phonological awareness have been shown to contribute significantly to reading (Engen and Høien, 2002), their influence may vary for children at different stages (Muter et al., 1997).

The separation of two phonological awareness abilities—manipulation and detection—has been extensively studied (Bentin, 1992). In general, detection abilities develop earlier than manipulation abilities, especially sound deletion (Anthony et al., 2003), and detection abilities have been shown to be the foundation of manipulation abilities (Bryant et al., 1990).

In Chinese, syllable-level phonological awareness has been found to be an important predictor of reading and for distinguishing children with and without reading difficulties (e.g., Lei et al., 2011). However, the importance of phonological awareness changes by age, especially during the preschool and primary school ages. For instance, Li et al. (2012) examined predictions of syllable deletion and rime detection for the Chinese character recognition of children in kindergarten (second year) and primary school (first to third years). Their results revealed a distinct difference in the phonological awareness of the two groups. In the kindergarten group, syllable deletion served as a significant predictor of Chinese character recognition, but rime detection did not. On the contrary, for the primary school group, Chinese character recognition was significantly predicted by rime detection but not by syllable deletion.

Considering the nature of phonetic supporting systems in mainland China and Taiwan, in which the onsets and vowels are emphasized separately, and the distinct contributions of phonological awareness to Chinese character reading in these two regions, the phonetic systems are closer to the onset-rime-level phonological awareness than to syllable-level phonological awareness, which has been the focus of previous studies (e.g., Siok and Fletcher, 2001). Therefore, training in syllable-level phonological awareness may not fully reflect the benefits of the phonetic supporting systems reviewed above. The phonological awareness training in Zhou et al.'s (2012) design mainly involved syllable-level phonological awareness. Their non-effective outcomes with regard to phonological awareness, Chinese character reading, and vocabulary knowledge may be due to this level instead of onset-rime-level phonological awareness. Therefore, this study aimed to investigate onset-rime-level phonological awareness training and its outcomes among children who learn Chinese without receiving phonetic supporting systems. Because Hong Kong primary school-aged children have not received relevant phonological awareness training, the focus on the detection of sound structures may be more suitable for measuring their phonological awareness, as shown in previous studies (e.g., Ho, 1997).

### Research aims

To examine the effects of phonological awareness training on individuals who learn Chinese without phonetic supporting systems, a fundamental ability level should be identified. This study aimed to examine the effects of phonological awareness training on the detection of onset-rime sounds for the reading-related abilities and Chinese character reading among Hong Kong children with dyslexia.

To test the effects of phonological awareness training, pre-test and post-test differences in relevant reading-related abilities, especially phonological awareness, and reading performance (e.g., Alexander et al., 1991) as well as the associations (contributions) of phonological awareness and reading performance (Bus and van Ijzendoorn, 1999) must be determined. Therefore, two research aims were addressed. The first aim was to compare phonological awareness, other reading-related abilities (i.e., orthographic knowledge and rapid naming), and character reading before and after the phonological awareness training of the experimental and control groups. The second aim was to compare the correlational patterns of the phonological awareness and character reading of the experimental and control groups before and after phonological awareness training.

Given the significant relationships of other reading-related abilities, such as rapid naming and orthographic knowledge, and Chinese character reading (Li et al., 2012), we included these two abilities in the present study. In terms of the strong association between phonological awareness and rapid naming in Chinese (e.g., Pan et al., 2011), it is believed that rapid naming is more likely to change with the improvement of phonological awareness. In contrast, phonological awareness and orthographic knowledge are separate in Chinese. In terms of Wang and Yang's (2016) research on how temporal processing influences reading-related abilities, only visual temporal processing could significantly predict orthographic knowledge, whereas only auditory temporal processing could significantly predict phonological awareness. This finding implies the modality-specific phenomenon between phonological awareness and orthographic knowledge in Chinese. Therefore, the involvement of orthographic knowledge was also considered as a control. That is, the improvement of phonological awareness should be independent and should not influence orthographic knowledge.

## Materials and methods

### Participants

Fifty-six Hong Kong children from the 3rd to 6th grades were recruited. Two-thirds of the children had been officially identified as dyslexic by the local education authorities. The other one-third of the children, who were considered high risk for dyslexia (i.e., at the second tier in the three-tier model for intervention and identification in Hong Kong), revealed a similar level of reading abilities as those who were identified as dyslexic. The reason for including high-risk dyslexic children was to reach a sufficient sample size for statistical power.

Two approaches were implemented to ensure the consistency of officially identified and high-risk dyslexic children. First, the officially identified and high-risk dyslexic children were randomly but separately placed into a control group (*N* = 28) and an experimental group (*N* = 28), with the groups matched mainly by age. There were 19 dyslexic and nine high-risk dyslexic children in the control group and 19 dyslexic and nine high-risk dyslexic children in the experimental group. Additionally, the children's performance on all demographic information, reading-related abilities, and reading performance was tested, and the results revealed that the differences on all variables were non-significant (*p* > 0.05). Furthermore, the criteria for participant selection in this study included normal or corrected-to-normal vision and hearing and the absence of intellectual and developmental disabilities.

In this study, the participants with dyslexia, including officially identified and high-risk children, were expected to perform at least one standard deviation below their respective age means on the Chinese word reading subtest of the Hong Kong Test of Specific Learning Difficulties in Reading and Writing (HKT-SpLD) (Ho et al., 2000a). This criterion for selecting children with dyslexia has been used by many previous studies (e.g., Berninger et al., 1998, 2008; Gallagher et al., 2000; Nielsen et al., 2016). The characteristics of the two groups of participants are shown in Table 1.

**Table 1:** Characteristics of the two groups of participants.

	**Reliabilities (Cronbach's α)**	**Experimental group (*****N*** = **28)**		**Control group (*****N*** = **28)**		***T***
		**Mean**	**SD**	**Mean**	**SD**	
Ages		99.82	12.77	97.21	12.01	0.25
IQ		9.45	1.13	9.52	1.06	0.44
Character reading (0–200)	0.81	47.96	4.76	47.11	5.04	0.66
Onset awareness (0–20)	0.67	8.86	2.61	7.54	2.91	1.79
Rime awareness (0–20)	0.62	9.50	2.85	9.54	2.77	−0.05
Lexical tone awareness (0–15)	0.72	4.86	1.86	4.68	1.54	0.39
Rapid naming (0–100)	0.72	30.55	4.44	30.86	4.40	1.29
Orthographic knowledge (0–34)	0.76	19.29	4.40	17.68	4.94	1.29

### Measurements

Measurements of non-verbal IQ, phonological awareness, rapid automatized naming, morphological awareness, and word reading were administered to all groups of participants before and after the training sessions. Participants were tested individually and within their own group over a few sessions depending on how long they could focus. The participants were provided with 5-min breaks after being tested for 20 min. They were assessed over two or more sessions on separate days. The measures were short, fast-paced and varied to maintain the children's motivation level and interest. All of the children's parents provided written informed consent to participate in this study. Ethical approval for the research was obtained from the Human Research Ethics Committee at the Education University of Hong Kong.

#### Non-verbal IQ test

Raven's Standard Progressive Matrices–Parallel is a standardized test of non-verbal IQ that consists of 60 items of increasing difficulty. Each item consists of a target visual matrix with one missing part. The children were asked to select from six to eight alternatives to fill a missing patch in a visual matrix. The Taiwanese norm for this test was established by the Chinese Behavioral Science Co., and the test-retest reliability was 0.81 within a 35-day period (Chen and Chen, 2006).

#### Character reading test

Huang (2001) developed the Chinese Character Recognition Scale, an individual test in which children are asked to pronounce every word as quickly as possible. The task arrangement is 20 lines; each line involves 10 Chinese words, for a total of 200 Chinese words in this task. Only the number of words answered correctly was counted as the final score of this test. The test-retest reliability was 0.94 within a 4 week period.

#### Phonological awareness test

Phonological awareness was measured with a three-part sound oddity task (for onset awareness, rime awareness, and tone awareness), which was adapted from Deacon and Kirby's (2004) rime oddity task. In each trial of each part, the children were asked to select by saying aloud which of three orally presented words differed from the others by one sound. The tester scored the participants' oral responses immediately after they were produced. Each part had 20 items, and two practice trials were performed before each part.

#### Rapid naming test

This task was taken from the Rapid Automatized Naming Test (Tzeng et al., 2011). In this study, only digit rapid naming was used because it has been demonstrated to be a powerful contributor to reading in Chinese (Georgiou et al., 2008). The participants were asked to name digits from left to right as quickly and as accurately as possible. The total time needed to name all 40 stimuli (i.e., digits) was recorded and analyzed.

#### Orthographic knowledge test

Two tests were involved: a semantic radical task and a phonetic radical task. The semantic radical task was taken from Hung and Fang (2006a). In this task, 17 multiple-choice items are constructed from characters with semantic radicals with different frequencies, different positions, and deformation. The participants were asked to choose the character that did not have relevant meaning. For instance, four stimuli were 鐵 [iron], 銅 [copper], 銹 [rust], and 信 [believe]. The participants were asked to select 信 [believe] because only this character contains the semantic radical “
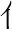
” [human], which is related to humans, whereas the other three characters contain “
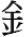
” [gold], which is related to metal. The suitable grade levels for this standardized measurement are from 3rd grade to 9th grade. This task is used to test participants' radical information knowledge and positional information knowledge for semantic radicals. The phonetic radical task is taken from Hung and Fang (2006b). In this task, 17 multiple-choice items are constructed from exceptional characters with phonetic radicals with different positions and different phonics. The participants are asked to choose one that is pronounced similarly to the target character. For instance, the participants were provided with 妙/miao4/ and asked to choose 秒/miao3/ from 秋/qiu1/, 桐/tong2/, and 妤/yu2/. The suitable grade levels for this standardized measurement are from 3rd grade to 9th grade. This task is used to test participants' radical information knowledge and positional information knowledge of phonetic radicals.

### Training sessions

Based on the design of phonological awareness training in Torgesen et al. (1992), this training program included two sessions: warm-up and training. The training procedure took ~3 weeks, with 15 daily sessions lasting ~20 min each. This training was implemented in small groups, each with two trainers and four to six participants. The participants were conveniently assigned into small groups based on their schools, and they stayed with the same group for all training sessions. The pre-test was administered immediately before the first session of training, and the participants received the post-test within 5 days after training.

During the warm-up period, the trainers played a variety of first- and last-sound games with the children to establish rapport and to gradually move the children from familiar to relatively unfamiliar activities. In both the first- and last-sound games, the participants were introduced to three interesting sounds (e.g., meow, growl, chirrup) sequentially with a fixed tempo of music. They were asked to pronounce the one chose by the trainers. These activities were also used to begin to focus the children's attention on the separate sounds within words.

After the warm-up period, the students were first taught to identify and pronounce the beginning or ending sounds in each character. The changes of lexical tones were also emphasized by the trainers because of the importance of lexical tone awareness to Chinese reading (e.g., Wang et al., 2017). For instance, both 花 (flower/hua1/) and 掛 (hitch /gua2/) exist in the song Little Sun; they were selected and paired to teach the students about the ending sounds. Furthermore, the children were taught to pronounce all the sounds of a Chinese character separately (analysis), and they were finally taught to pronounce words after hearing their phonemes presented in sequence (synthesis).

After the introduction of the sound identification, the students were given a famous song in each training session. All these songs were popular and age appropriate for the participating children, and most of the children confirmed being familiar with these songs. They were asked to sing the song together by following the music without the lyrics after the trainers' demonstration. The trainers then provided the lyrics and pointed to the vocabulary words one by one. In this procedure, the words with targeted Chinese characters were highlighted. The means of selecting the target characters was based on the suggested Learning Levels provided by Poon and Hong (2003). Considering the children's poor reading achievement in school, only characters belonging to the Learning Levels of first to fourth grade in primary school were selected. The numbers of the target character varied (from seven to thirteen) because it is necessary to match those characters with the same pronunciation in different parts of Chinese characters' sounds (please see the **Annex** for the examples used in the training sessions). However, all three parts of the Chinese characters' sounds (i.e., beginning sound, ending sound, and lexical tone) were not controlled due to the limited characters in the lyrics of each song. The students were then shown cards with words containing the targeted Chinese characters and pictures showing the meanings of the vocabulary words.

The training providers were undergraduate research assistants from Hong Kong. All had received at least two training sessions and two teaching observations by the authors. To ensure training quality, the authors provided the learning objectives and recommended activities, and discussions were compulsory when preparing all teaching sessions. In addition, the authors arranged one trial lesson for each trainer to ensure that the training provided reached the expected fidelity level. A checklist with each step of the training session, in which the brief introductions of activities in different periods were given, was provided to the trainers to help ensure that their training had identical crucial elements for this study and to enhance the fidelity of the intervention. In this case, the trainers' time control, content delivery, teaching material presentations, and volume of voice were observed by the researchers.

In contrast to the participants in the experimental group who received phonological awareness training, the participants in the control group received no intervention during the experiment in this study. They simply returned home when the experiments implemented and did no activity related to reading. Furthermore, to reduce the possible confounding factors, the participants in both the experimental and control groups received pre- and post-tests within the same periods of time. To support the ethical considerations of this study, all of the control group participants were provided with identical interventions as the experimental group participants after data collection.

### Data analysis

To achieve the research aims of this study, a number of statistical methods were introduced. For the first research aim, the comparisons of phonological awareness, other reading-related abilities (i.e., orthographic knowledge and rapid naming), and character reading before and after the phonological awareness training of the experimental and control groups were analyzed by paired-sample *t*-tests for intragroup differences and an independent analysis of covariance (ANCOVA) for intergroup differences. In addition, the comparisons the correlational patterns of the phonological awareness and character reading of the experimental and control groups before and after phonological awareness training, which is the second research aim, were analyzed by partial correlation analyses along with the revised Fisher's Z procedure with two-tailed correlation.

## Results

**Figure 1:**
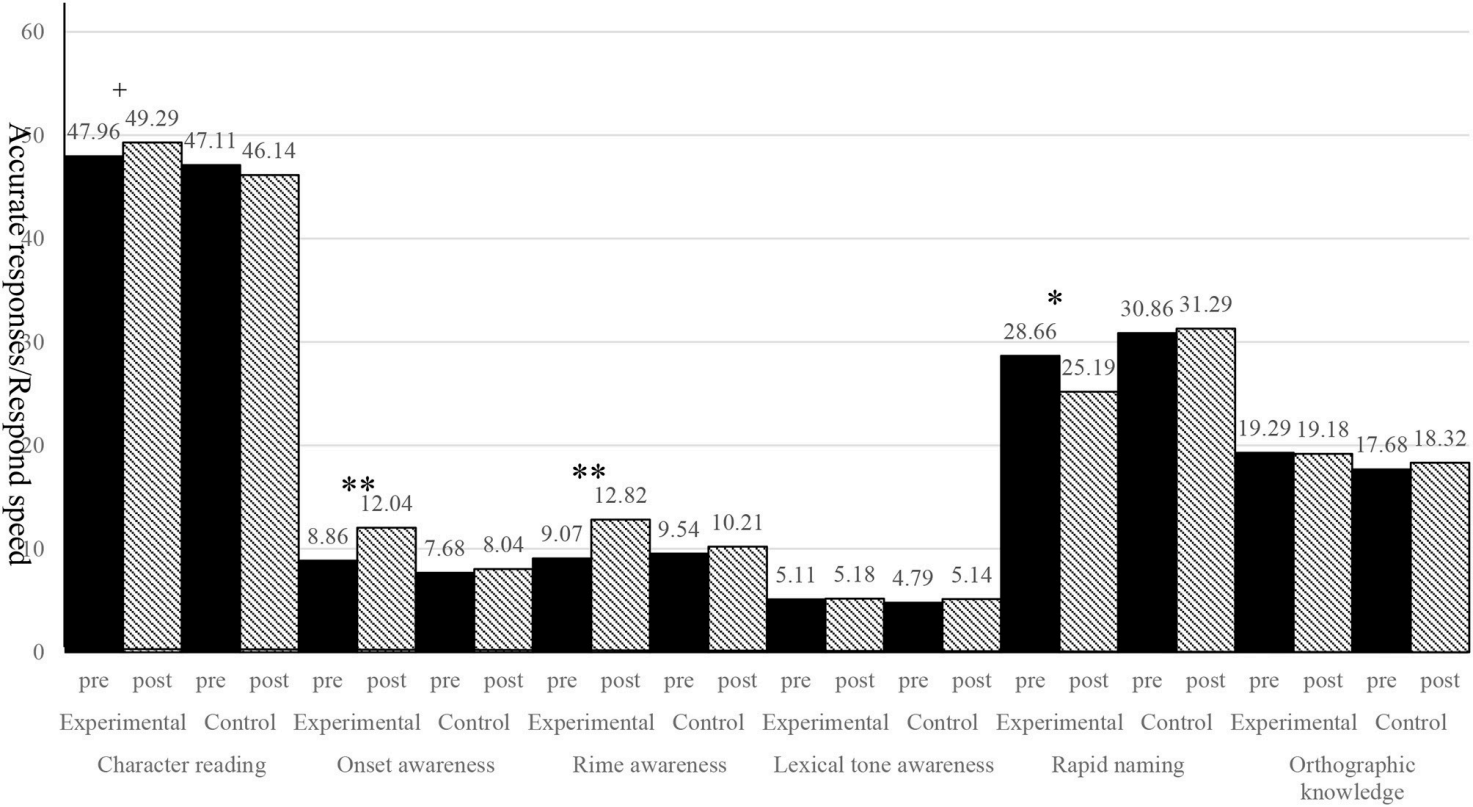
The comparisons of phonological awareness, rapid naming, orthographic knowledge, and character reading before and after the phonological awareness training for experimental and control groups respectively. + *p* ≤ 0.06, ^*^p < 0.05, ^**^p < 0.01.

With regard to the first research aim, intragroup improvements were examined via paired-sample *t*-tests to compare performance on phonological awareness, rapid naming, orthographic knowledge, and character reading before and after the phonological awareness training of the experimental and control groups. The results are shown in in Figure 1.

Our results revealed that significant differences were found in onset awareness [*t*_(27)_ = −6.09, *p* < 0.01, Cohen's *d* = −1.29], rime awareness [*t*_(27)_ = −7.74, *p* < 0.01, Cohen's *d* = −1.68], and rapid naming [*t*_(27)_ = 2.54, *p* < 0.05, Cohen's *d* = 0.64] before and after training for the experimental group, whereas no significant difference was found in any task before and after training for the control group [onset awareness: *t*_(27)_ = −0.83, *p* > 0.05, Cohen's *d* = −0.14; rime awareness: *t*_(27)_ = −1.04, *p* > 0.05, Cohen's *d* = −0.26; rapid naming: *t*_(27)_ = −0.44, *p* > 0.05, Cohen's *d* = −0.09]. Chinese character reading showed only marginal positive difference [*t*_(27)_ = −1.95, *p* = 0.06, Cohen's *d* = −0.29) between before and after training performance of the experimental group. Among these findings, the level of difference was greatest for rime awareness.

To address another aspect of the first research aim, an independent analysis of covariance (ANCOVA) was used to compare group differences after the onset-rime-level training sessions in phonological awareness, rapid naming, orthographic knowledge, and character reading with the covariates of the participants' IQ and pre-test scores. ANCOVAs with the controlling pre-test scores were used to analyze the data per Dimitrov and Rumrill's (2003) suggestion to “reduce the error variance and eliminate systematic bias.” The results are presented in Table 2.

**Table 2:** Comparisons of phonological awareness, other reading-related abilities, and Chinese character reading of experimental and control groups and typically developing children after training with IQ and controlling pre-test scores.

	**Experimental group (*****N*** = **28)**		**Control group (*****N*** = **28)**		***F*_(1, 54)_**	**Partial η^2^**
	**Mean**	**SD**	**Mean**	**SD**		
Character reading	49.29	4.39	46.14	5.24	7.23^*^	0.12
Onset awareness	12.04	2.32	8.04	2.36	38.42^**^	0.42
Rime awareness	12.82	1.89	10.21	2.35	22.59^**^	0.30
Lexical tone awareness	5.18	1.63	5.14	1.41	0.25	0.01
Rapid naming	25.19	5.19	31.29	5.61	14.39^**^	0.21
Orthographic knowledge	19.18	3.92	18.32	4.90	0.01	0.00

* *p < 0.05*,

** *p < 0.01*.

In comparisons of the post-training performances of the experimental and control groups, experimental groups significantly outperformed control group in Chinese character reading, onset awareness, rime awareness, and rapid naming. The difference was greatest for rime awareness.

To achieve the second aim, considering the limited sample size of each group, partial correlations were introduced to compare the correlational patterns of phonological awareness and character reading of the experimental and control groups before and after phonological awareness training. The results are shown in Table 3.

**Table 3:** Partial correlations of phonological awareness and Chinese character reading of the two groups before and after training with controlling IQ.

		**1**	**2**	**3**	**4**
Experimental group	1. Character reading	–	0.02	0.05	0.05
	2. Onset awareness	0.21	–	0.15	0.38^+^
	3. Rime awareness	0.46^*^	−0.15	–	−0.11
	4. Lexical tone awareness	−0.27	0.19	−0.05	–
Control group	1. Character reading	–	0.23	0.22	−0.13
	2. Onset awareness	−0.06	–	−0.20	0.01
	3. Rime awareness	0.14	−0.04	–	−0.06
	4. Lexical tone awareness	−0.02	−0.03	0.13	–

*The numbers highlighted in gray are for after training*.

+ *p < 0.06*,

* *p < 0.05*.

Our results indicated that the relationships between different components of phonological awareness and Chinese character reading were stable, except that the difference in rime awareness and Chinese character reading changed from non-significant (before training) to significant (after training).

To more precisely compare whether the correlations of the three components of phonological awareness and character reading of the two groups before and after training were similar or different, we used the method of Silver et al. (2004), which was a modified version of Dunn and Clark's z using a back-transformed average Fisher's Z procedure with two-tailed correlation.

Our results revealed no significant difference in the relationship of Chinese character reading and onset awareness between the pre-test and the post-test for either the experimental group (*z* = −0.80, *p* = 0.21) or the control group (*z* = −1.35, *p* = 0.09). We found a similar pattern for the relationship of Chinese character reading and lexical tone awareness between the pre-test and the post-test for both the experimental group (*z* = 1.69, *p* = 0.95) and the control group (*z* = 0.44, *p* = 0.67). However, although the difference in the relationship of Chinese character reading and rime awareness between the pre-test and the post-test was not significant for the control group (*z* = −0.29, *p* = 0.39), a significant difference was found for the experimental group (*z* = −1.79, *p* = 0.04).

Although the results of both research aims are discussed above, it is important to note that the difference in Chinese character reading between the pre- and post-test in the experimental group is only slightly significant. That is, the effectiveness of our onset-rime-level phonological awareness training does not transfer as well to reading performance as expected. Given the findings of Ho et al. (2000b), in which dyslexic children with average ages of seven to 10 showed significantly poorer performance for both onset and rime detection than their age-matched peers, there may be an influence of the participants' demographic data.

To further examine the crucial factors in the improvement of Chinese character reading in the experimental group, a multiple stepwise regression was introduced. All demographic information and pre-test performance were included as predictors. The results revealed that only the predictions of age, IQ, and pre-test character reading performance were significant for the difference in the pre- and post-test on Chinese character reading. Among these three factors, the importance of age was far higher than the importance of IQ and pre-test character reading performance [age (*R*^2^ changed = 0.33, *p* = 0.001); pre-test character reading (*R*^2^ changed = 0.17, *p* = 0.009); IQ (*R*^2^ changed = 0.11, *p* = 0.016)]. Therefore, to further investigate our result, separate examinations of the experimental group participants by dividing them into different age levels is necessary.

The age of 10 was treated as the cut-off age for two reasons. Theoretically, many previous studies have indicated that children's cognitive abilities develop rapidly until ~10 years old (e.g., Anderson, 2002; Tillman et al., 2008). This view was supported by Ho et al. (2000a) findings, in which the age of 10 seemed to be the ceiling for identifying dyslexic children's poor onset-rime-level phonological awareness. Furthermore, when distinguishing between pre-test and post-test character reading in this study, four of the 24 (16.7%) children whose differences were above the mean were older than 10 years old. On the contrary, for those whose differences between pre-test and post-test character reading were below the mean, 19 of 32 (59.3%) were older than 10 years old.

We were surprised to find a clear pattern of differences in the impact of phonological awareness training at different ages. That is, participants younger than 10 years old in the experimental group were observed to benefit more from phonological awareness training in terms of Chinese character reading, onset awareness, rime awareness, and rapid naming, whereas those older than 10 years old were observed to receive fewer benefits from this training, especially for Character reading and rapid naming. The details of the pre-test and post-test comparisons of the participants in the experimental group who were older and younger than 10 years old are shown in Table 4.

**Table 4:** Comparisons of reading and reading-related abilities of before and after training for experimental groups below and above 10 years old.

		**Before training**		**After training**		***t*_(15/11)_**	**Cohen's *d***
		**Mean**	**SD**	**Mean**	**SD**		
Below 10 years old (*N* = 16)	Character reading	47.19	4.31	50.31	2.89	−3.48^**^	−0.85
	Onset awareness	8.63	2.96	12.06	2.79	−4.24^**^	−1.19
	Rime awareness	8.63	2.45	12.75	2.02	−6.75^**^	−1.83
	Lexical tone awareness	4.75	1.91	4.94	1.81	−0.64	−0.10
	Rapid naming	28.16	6.51	22.32	3.87	3.18^**^	1.09
	Orthographic knowledge	18.94	4.78	18.56	4.00	0.23	0.09
Above 10 years old (*N* = 12)	Character reading	49.00	5.31	47.92	5.68	2.17	0.20
	Onset awareness	9.17	2.12	12.00	1.60	−4.82^**^	−1.51
	Rime awareness	9.67	2.61	12.92	1.78	−4.12^**^	−1.45
	Lexical tone awareness	5.58	1.44	5.50	1.38	0.25	0.06
	Rapid naming	29.33	4.65	29.01	4.24	0.19	0.07
	Orthographic knowledge	19.75	3.98	20.00	3.81	−0.17	−0.06

** *p < 0.01*.

In sum, our results revealed that the onset-rime-level phonological awareness training in this study can be beneficial for the phonological awareness of children with dyslexia who learn Chinese without receiving phonetic support systems and that the transfer effect to rapid naming and Chinese character reading can only be found in those younger than 10 years old.

## Discussion

This study aimed to examine the effects of phonological awareness training on the detection of onset-rime sounds and reading-related abilities and Chinese character reading among Hong Kong children with dyslexia. Two research aims were addressed. Overall, this project found that onset-rime-level phonological awareness training had a major impact on the reading performance of Hong Kong children with dyslexia.

With regard to the first research aim, the comparison of the pre-test and post-test scores of dyslexic children in the experimental group showed that they mainly benefitted from the onset-rime-level phonological awareness training in terms of onset awareness, rime awareness, and rapid naming. Although the significant influences on onset awareness and rime awareness were as expected based on the findings of previous studies in Hong Kong (e.g., Ho and Bryant, 1997), the significant influence (or the lack thereof) of onset-rime-level phonological awareness training on rapid naming and lexical tone awareness had to be addressed.

Our results revealed significant improvement in rapid naming after the phonological awareness training. Rapid naming, which refers to the ability to name highly familiar visual stimuli very quickly (Georgiou et al., 2008), is also regarded as a kind of phonological processing (Wagner and Torgesen, 1987). Although the independence of rapid naming from phonological awareness has been proposed and justified for some time (e.g., Wolf et al., 2000), the strong associations of rapid naming and phonological awareness with reading, especially for poor readers, have remained ambiguous (e.g., McBride-Chang and Manis, 1996; Kirby et al., 2003).

In this study, the focus on the type of phonological awareness that develops earlier (i.e., detection) might explain the association between phonological awareness training and rapid naming. However, the association of the detection of onset-rime sounds and rapid naming can vary as familiarity with the phonetic system increases (e.g., Shu et al., 2008).

Our results also revealed that the difference in lexical tone awareness between pre-test and post-test scores (before and after phonological awareness training) was not significant. In Chinese, lexical tones are pitch patterns that influence different levels of language processing and that are particularly related to the linguistic domain (Gandour, 1978), ranging from segmental analysis of words to supra-segmental processing of sentence structure. Considering the unsystematic nature of Chinese characters, lexical tone awareness has been thought to potentially help Chinese readers distinguish between homophones (Tong et al., 2011) and determine the relationships of the pronunciations of whole characters and phonetic radicals (Chen et al., 2003).

As previously discussed, the differences between lexical tone awareness and onset awareness and rime awareness were unsurprising (Shu et al., 2008). In addition, the potential need for more comparisons of different or similar lexical tones to provide effective lexical tone awareness training (e.g., Wang et al., 1999, 2003), which the phonological awareness training in this study did not emphasize, was expected.

When addressing the first research aim of this study, significant differences were found in Chinese character reading, onset awareness, rime awareness, and rapid naming between the post-test scores of the participants in the experimental group and those in the control group. Most of the results were in line with the results we found by comparing the pre-test and post-test scores of the participants in the experimental group for the first analysis of the first research aim via paired-sample *t*-tests. The impact of phonological awareness training on Chinese character reading was clearer here, but only a marginal effect in the pre-test and post-test comparison of the experimental group was found.

Our results of the effectiveness of phonological awareness training for Chinese children who learn Chinese without receiving phonetic support systems were slightly different from those of previous studies. In our study, the comparisons of post-test character reading and phonological awareness between the experimental and control groups revealed significant improvements due to phonological awareness training, which were not found in Zhou et al.'s (2012) study. This difference may be due to the distinct levels of phonological awareness and the obvious gap in the participants' ages.

Hong Kong children's sensitivity to onset, rime, and lexical tone has been found to develop by age seven (Ho and Bryant, 1997). This onset-rime-level phonological awareness occurs far after syllable-level phonological awareness (Ziegler and Goswami, 2005). Therefore, since the participants in Zhou et al.'s (2012) study had an average age of 74 months, syllable-level phonological awareness, which was the focal point in their study, may be already or close to mature at this timing. This may be the reason for the non-significant improvements from the phonological awareness training in their study.

Regarding the correlation analysis conducted for different components of phonological awareness and Chinese character reading, a significant change was found in the correlation of rime awareness and Chinese character reading. This finding is partially consistent with the findings of previous studies (e.g., Bus and van Ijzendoorn, 1999). In terms of the greater importance of rime awareness in less-transparent orthographies such as English and Chinese (Goswami, 2002), the significant impact of phonological awareness training on rime awareness could be expected to improve Chinese character reading.

Finally, our findings that 10 was the average cut-off age for maximizing beneficial earning from onset-rime-level phonological awareness training in this study echo the finding that the influence of age on the effects of onset-rime-level phonological awareness training might reflect the developmental ceiling of auditory temporal processing, which occurs at ~10 years old (e.g., Hautus et al., 2003). Auditory temporal processing has been found to contribute significantly to the phonological awareness of Chinese children with and without dyslexia in early and middle primary school (Wang and Yang, 2016). This clear pattern also partially echoed recent evidence on the less significant prediction of phonological awareness on reading performance after 10 years old (e.g., Song et al., 2015).

The present study has at least four limitations. First, as previously mentioned, phonological awareness exists on three levels: the syllable level, the onset-rime level, and the phoneme level (Gillon, 2004). However, this study focused only on onset-rime-level phonological awareness. Although our findings indicated the preliminary effects of this training, they might be limited because we chose a specific level of phonological awareness. Additionally, considering the developments of different types of phonological awareness (Anthony and Francis, 2005), the differential effects of phonological awareness on children of different ages should be further investigated in future research.

Second, phonological awareness includes at least two abilities: detection and manipulation (Bentin, 1992). Although detection abilities have been shown to be the foundation of manipulation abilities (Bryant et al., 1990), manipulation abilities, especially deletion, have proven to be great predictors of both Chinese and English word reading (Chow et al., 2005). Since the limited phonological information capabilities of Hong Kong children with dyslexia were considered, only detection ability was involved in this study. Training in more complex phonological awareness abilities may have different effects on reading and reading-related abilities.

Third, our design involved the meaning of the vocabulary. This may be due to some confusion regarding the purity and fidelity of our phonological awareness training. Although we adapted this training from Torgesen et al.'s (1992) design and did not emphasize this issue, the intention of involving the vocabulary is to enhance motivation and connect our training to the children's lives. However, we cannot avoid the possible influence of the semantic aspect in this training. Therefore, a training design with greater purity of phonological awareness is necessary in the future.

Finally, the finding regarding the difference in training effects on children older and younger than 10 years old is surprising and was not expected in our original design. The limited sample sizes of each age group (16 children younger than 10 years old and 12 children older than 10 years old) in the experimental group may have affected the statistical accuracy of our results. A further experiment could attempt to replicate the results of the influence of age on the effects of phonological awareness training.

In addition to the developmental issues caused by age, a life-experience difference may also occur among children of different ages. For instance, the older children with dyslexia may have faced more failure experience, which may cause learned helplessness and low self-esteem and may substantially affect their responses to any training (Burden and Burdett, 2005). Furthermore, although the songs used in our training program were all popular and age appropriate, different participants' familiarity with different songs varied. For more familiar songs, people may expend less working memory loading on melody and lyrics and have more room to identify the phonological components, as suggested by Cognitive Load Theory (Sweller et al., 2011). In this case, the older children may have been less familiar with the songs than the younger children were, which may have influenced the results in addition to developmental issues.

## Conclusion

Despite these limitations, this study preliminarily demonstrated the impact of phonological awareness training on the detection of onset-rime sounds. Our results revealed that such training could be effective for phonological awareness and Chinese character reading in Chinese children with dyslexia, but this impact was limited by age. The major influence of phonological awareness training was found to be transferable from phonological awareness, especially rime awareness, to Chinese character reading if the dyslexic children were younger than 10 years old, whereas the effects of phonological awareness training were limited and significant for onset awareness and rime awareness for children older than 10 years old.

On a theoretical level, our findings may fill a research gap with regard to the importance of phonological awareness (onset-rime level) for Chinese children with dyslexia who learn Chinese without phonetic support systems. On a practical level, if subsequent research supports our conclusions, this study can be extended to provide another approach to Chinese learning for those suffering from reading difficulties (i.e., dyslexia) who are not responding to the usual approach in their region.

## Author contributions

LW is the sole author of this research article and his task involves conception or design of the work, data analysis and interpretation, drafting the article and final approval of the version to be published.

### Conflict of interest statement

The author declares that the research was conducted in the absence of any commercial or financial relationships that could be construed as a potential conflict of interest.

## Annex

Examples of vocabularies contain target Chinese characters in the training sessions.

1. The same beginning sounds
  (1) “太陽”(/tai4 yang2/, sun) and “天空” (/tian1 kong1/, sky)
  “太” (/tai4/, extremely) and “天” (/tian1/, sky)
  (2) “朋友” (/peng2 you3/, friend) and “害怕” (/hai4 pa4/, fear)
  “朋” (/peng2/, friend) and “怕” (/pa4/, fear)

2. The same ending sounds
  (1) “紅花” (/hong2 hua1/, red flower) and “說話” (/shuo1 hua4/, speak)
  “花” (/hua1/, flower) and “話” (/hua4/, speech)
  (2) “宇宙” (/yu2 zhou4/, universe) and “逃走” (/tao2 zou4/, escape)
  “宙” (/zhou4/, eternity) and “走” (/zou4/, walk)

3. The same lexical tones
  (1) “尊敬” (/zun1 jing4/, respect) and “依舊” (/yi1 jiu4/, still)
  “尊” (/zun1/, senior) and “依” (/yi1/, depend on)
  (2) “秘密” (/mi4 mi4/, secret) and “人物” (/ren2 wu4/, figure)
  “密” (/mi4/, secret) and “物” (/wu4/, object)
